# Response of Surface Soil Hydrology to the Micro-Pattern of Bio-Crust in a Dry-Land Loess Environment, China

**DOI:** 10.1371/journal.pone.0133565

**Published:** 2015-07-24

**Authors:** Wei Wei, Yun Yu, Liding Chen

**Affiliations:** State Key Laboratory of Urban and Regional Ecology, Research Center for Eco-Environmental Sciences, Chinese Academy of Sciences, Beijing, China; Beijing Normal University, CHINA

## Abstract

The specific bio-species and their spatial patterns play crucial roles in regulating eco-hydrologic process, which is significant for large-scale habitat promotion and vegetation restoration in many dry-land ecosystems. Such effects, however, are not yet fully studied. In this study, 12 micro-plots, each with size of 0.5 m in depth and 1 m in length, were constructed on a gentle grassy hill-slope with a mean gradient of 8° in a semiarid loess hilly area of China. Two major bio-crusts, including mosses and lichens, had been cultivated for two years prior to the field simulation experiments, while physical crusts and non-crusted bare soils were used for comparison. By using rainfall simulation method, four designed micro-patterns (i.e., upper bio-crust and lower bare soil, scattered bio-crust, upper bare soil and lower bio-crust, fully-covered bio-crust) to the soil hydrological response were analyzed. We found that soil surface bio-crusts were more efficient in improving soil structure, water holding capacity and runoff retention particularly at surface 10 cm layers, compared with physical soil crusts and non-crusted bare soils. We re-confirmed that mosses functioned better than lichens, partly due to their higher successional stage and deeper biomass accumulation. Physical crusts were least efficient in water conservation and erosion control, followed by non-crusted bare soils. More importantly, there were marked differences in the efficiency of the different spatial arrangements of bio-crusts in controlling runoff and sediment generation. Fully-covered bio-crust pattern provides the best option for soil loss reduction and runoff retention, while a combination of upper bio-crust and lower bare soil pattern is the least one. These findings are suggested to be significant for surface-cover protection, rainwater infiltration, runoff retention, and erosion control in water-restricted and degraded natural slopes.

## Introduction

The deterioration of soil hydrological services, mainly including infiltration reduction and water erosion intensification, can aggravate the already-existing severe drought and land degradation in many dry-land regions (e.g., the Mediterranean area and Loess Plateau in China), particularly in the context of accelerated global warming [1–5]. The widespread bio-crust mosaics in the surface soil may play constructive roles in repairing soil hydrologic functions [6–8].

Bio-crusts in the uppermost millimeters of the topsoil, also known as "biological soil crusts (BSCs)", are common surface covers in many arid and semiarid environments [9–10]. These intertwined microorganisms mainly include mosses, lichens, green algae, micro-fungi, and other bacteria [11–13]. These spore species are an important and integral part of desert ecosystems, covering 40%-100% of the ground [14–15]. Compared to BSCs, however, higher vascular plant species usually cover less than 40% of the total surface area in these ecosystems [16]. Consequently, BSCs now are recognized as crucial elements that directly connect biotic and abiotic ingredients in the arid and semiarid landscapes [17], being significant in soil carbon sequestration, slope stability, desertification prevention and plant recruitment [18–19].

The surface coverage and spatial distribution of BSCs can strongly affect soil physiochemical attributes associated with multiple hydrologic properties, because they are important for the coupled eco-hydrological processes in the semiarid patchy plant-free interspaces [15, 20–21]. Compared with the role of physical crusts (PCs, formed by wind and water forces at the soil surface), different BSCs may greatly affect soil physical structure and aggravate stability, water evaporation and organic matter [12–13]. With an increase in lichen coverage on a degraded hill-slope in Spain, a clear decline rate in the risk of runoff and erosion was observed [22]. The mean depth, surface roughness, and micro-relief under various BSCs species differ from each other [23]. Such surface features, however, are closely linked to water infiltration capacity, soil moisture storage, surface runoff, and water erosion processes, which influence the actual effects of soil and water conservation, micro-site improvement, and vegetation establishment [5, 24]. As a result, the widespread BSCs in the semiarid areas are considered as "desert biological carpets" or "ecosystem engineers" by ecologists, hydrologists, and environmentalists [9]. Due to these advantages over PCs, BSCs widely cultivated on a large scale, will greatly benefit environmental protection and erosion control in the arid and semiarid regions. The cultivation of surficial BSCs pattern and its related hydrological response and ecological services has become a focus of research in recent years [25].

However, controversies still remain regarding the response of surface soil hydrology to the spatial pattern of BSCs species. The relationship between BSCs, infiltration capacity and related runoff-erosive effects is unclear, although global studies across different ecosystems have been conducted [24, 26]. The captured relationship between infiltration and BSCs patterns by different scientists differed (e.g., positive, negative or neutral), resulting in a lack of consensus and misleading [12, 27–30]. For example, Xiao et al. [28] found that mosses and lichens in the Loess Plateau can reduce water infiltration and increase potential erosion rates through decreasing soil saturated conductivity, while another study in the same region declared that BSCs can increase soil fertility, porosity, aggregate stability and thus reduce soil erosion risks [14].

Moreover, although the dynamics and distribution of soil surface components (vegetation, soil crusts, and rock fragments) are confirmed as key factors in maintaining soil hydrological function on hill-slopes [30], previous studies mainly focused on higher vascular vegetation patterns (forests, shrubs, and grasses). Little attention was paid to the role of BSCs patterns at finer scales. How to regulate infiltration, runoff and soil loss by adjusting BSCs pattern remains unclear [12], which becomes a key issue to be highlighted. The semiarid loess area in China has long been suffering from severe drought and water erosion [2, 31].Surface BSCs, particularly mosses and lichens, are widely developed and the mean coverage of these species can reach approximately 70% [14]. This indicates that BSCs species and their spatial patterns are significant in the hydrologic and erosive conditions in this fragile area [12]. We have therefore selected this region for our BSC studies.

Specifically, we selected a grass covered hill-slope in a typical loess area for plot construction and BSCs pattern design. Rainfall simulations were used as supplemental tools to study rainfall-runoff processes. Our main objectives were: (1) to compare and re-test the role of BSCs species in soil hydrologic attributes with PCs and bare soils, (2) to discuss the effect of major BSCs species on surface runoff and soil loss rates, and more importantly (3) to highlight how the micro-pattern of BSCs affects runoff and sediment generation.

## Materials and Methods

### Study area

**Fig 1:**
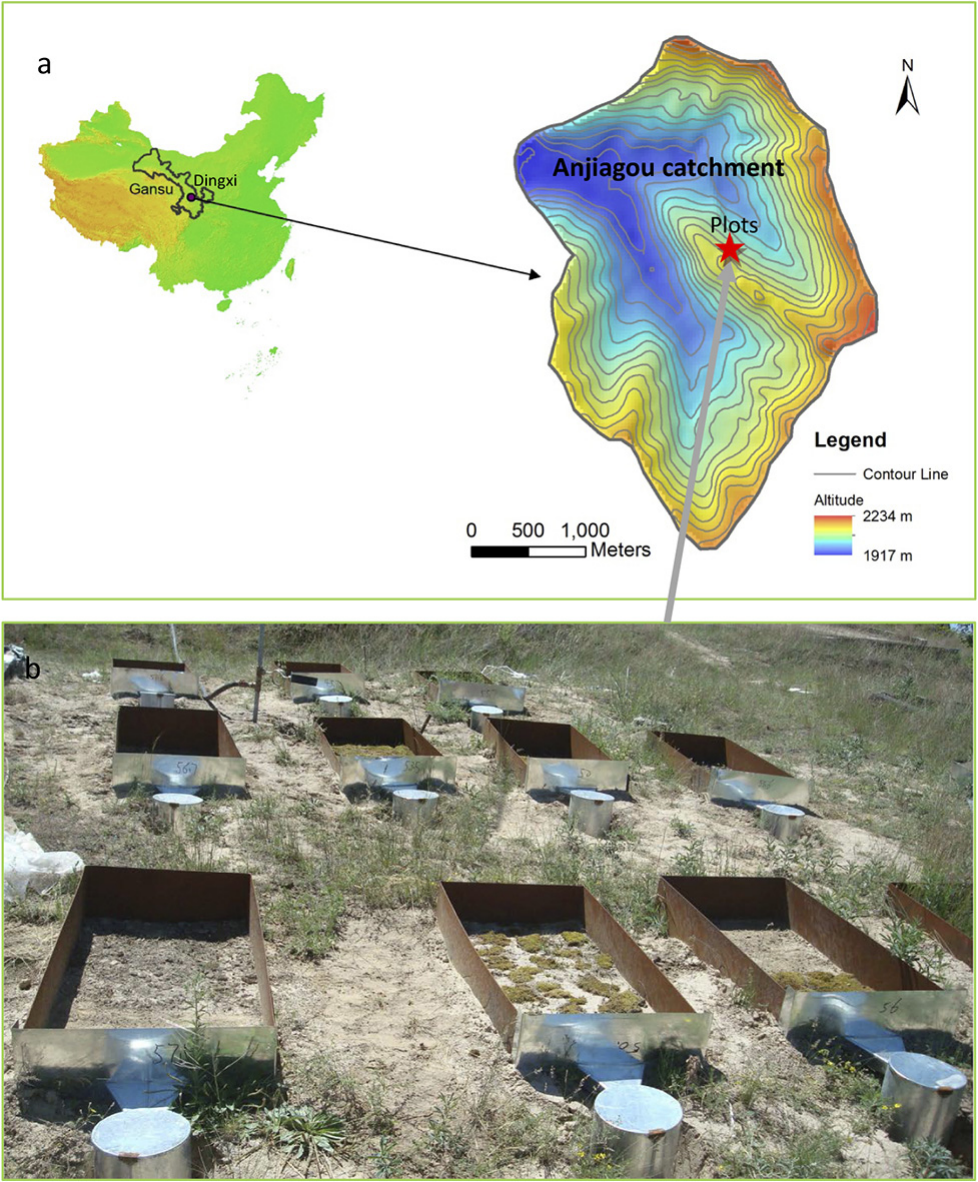
Location of the study area (a) and designed micro-plots (b).

Hydrological simulation experiments were conducted at the Anjiapo catchment (35°33'–35°35'N, 104°38'–104°41'E), a loess hilly region in Dingxi of Gansu province (Fig 1). There was no specific permissions were required for these locations and activities, because we have a trustful cooperation between our department and local institute. More importantly, the purpose of this research aims at conserving soil and water, reducing erosion and enhancing ecological restoration. Therefore, it is supported by the local authorities, the government and the local people. The field studies did not involve endangered or protected species. On the other hand, it will help to improve the degraded habitat and benefit the species.

This region belongs to a semiarid zone and is dominated by warm-humid summers and cold-dry winters. The mean annual precipitation is 421 mm/yr, of which nearly 80% occurs during the growing season, from May to September [32]. Rainstorms produce the majority of the annual rainfall, causing soil loss on the slopes. Based on meteorological station data, mean potential evaporation can reach 1515 mm per year. Local precipitation has experienced a slight decrease, while the temperature has continued to increase over the last several decades. This means that the local climate has become drier and warmer, which may further aggravate water restriction and hinder ecosystem restoration.

Local soil is developed from loess material, with the mean soil depths ranging from 40 to 60 m. The deepest soil layer in some areas can reach or exceed 100 m. Based on the international soil classification system, local soil is dominated by calcic Cambisol with a clay content of 33–42%, organic matter of 4–13g/kg, and a bulk density from 1.09 to 1.36g/cm^3^ within a 2 m soil depth [2, 31]. No groundwater is available for the different flora (e.g., spore species and high vascular vegetation), mainly due to the deep loess soil and severe drought. The limited rainfall is the only available water resource for all plants.

In general, the local climate and soil properties are more suitable for different grasses and BSCs species [14]. As a result of the "grain-for-green" program (the conversion of sloping cropland to forestland or grassland), "natural forest protection," labor service exports and other projects targeting enriching local farmers and protecting the environment, steep farming have been largely prohibited and croplands were returned to forests and shrubs, or left for natural succession [2–3, 31]. Under such conditions, soil disturbance can be minimized in most cases. Widespread BSCs, as a key component of the surface soil in this dry-land ecosystem, are highly developed. Based on *in situ* field investigation, mosses and lichens have been confirmed as the major types of BSCs in this region.

### BSC cultivation and micro-plot design

**Fig 2:**
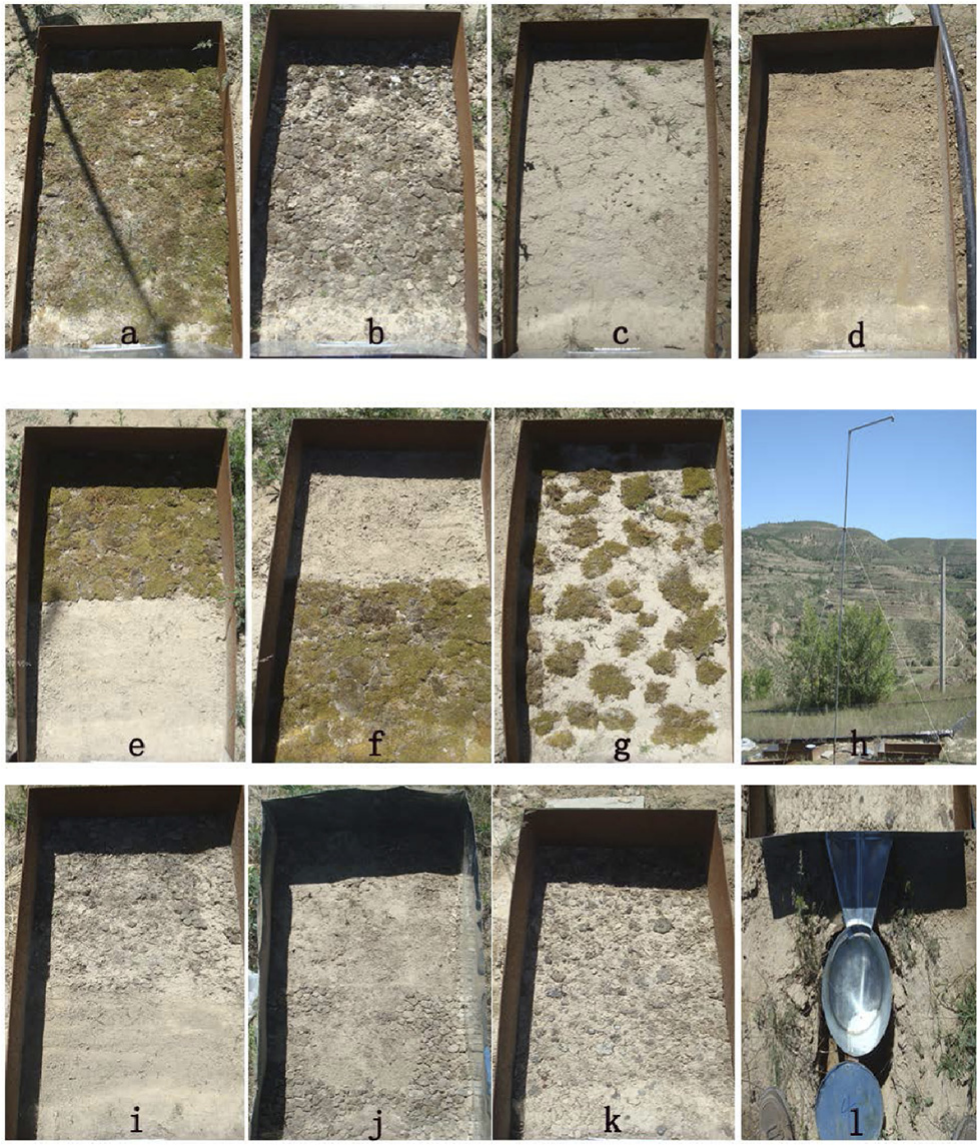
The designed micro-patterns of surface BSCs. Note: (a) fully-covered moss, (b) fully-covered lichen, (c) fully-covered physical crust, (d) non-crusted bare soil, (e) upper half mosses and lower half bare soil, (f) upper half bare soil and lower half moss, (g)scattered mosses with 50% coverage, (h) rainfall simulator, (i) upper half lichen and lower half non-crust soil, (j) upper half non-crust soil and lower half lichen, (k) scattered lichens with 50% coverage, and (l) runoff and sediment collector.

In July, 2011, a gentle slope (330°aspect and 8°gradient) covered by natural grasses in the Anjiagou catchment, was selected for micro-plot construction. In total, 12 micro-plots, with different BSCs types and arrangements, were established across the upper and middle sections of the selected slope (Fig 2). Among them, 8 plots were directly used for rainfall simulations, while the other 4 replicated plots (fully-covered mosses and lichens, PCs and non-crusted bare soil types) were designed only for soil sampling and data analysis. The specific area of each plot was designed as 0.5 m^2^ (0.5 m wide and 1.0 m long). All the plots were constructed and bounded by 30 cm high and 5 mm thick steel metal sheets that were inserted to half their height in the ground. These measures can keep plots stable and prevent any outside disturbance. Each micro-plot was equipped with a 50 L container to collect the expected runoff and sediment (Figs 1 & 2).

Before the plots were cultivated with BSCs, all natural weeds and other types of soil crusts were cleaned from the plots. The surface soil was flattened carefully to ensure the consistency of micro-landforms and soil features between the plots. The small gaps between soil inside the plot and the steel border were eliminated. The source materials and samples of the BSC species were collected from natural-grass slopes in the catchment. Before they were applied, the collected mosses and lichens were crushed and cultivated in the related plots with a crust density of 600 g/m^2^. After the BSCs were cultivated, approximately 500 ml of water was irrigated into each plot at one week intervals over natural rainfall events. When the BSCs and the topsoil had completely integrated two years later, the additional irrigation measures were stopped.

Four major types of fully-covered (to ensure the same coverage rate for better comparison) surface crusts were compared, i.e., mosses, lichens, PCs and non-crust bare soils (Fig 2). However, PCs and non-crust bare soil were mainly used for comparison with BSCs. PCs plots were kept naturally without any other human disturbance except artificial rainfall experiments. For non-crust bare soil plots, on the other hand, the surface 10 cm soil layer in the plots was loosened and then flatted carefully before each rainfall simulation, targeting for eliminating the temporarily formed PCs by raindrop impacts. Due to the relatively short simulation time (mostly less than 1 h), the influences of formed temporary PCs on infiltration and overland flow were not taken into account.

Moreover, four major micro-patterns of BSCs were included, i.e., fully-covered BSCs (FUCC pattern), scattered BSCs with 50% coverage (Scattered pattern), upper half BSCs with lower half non-crust bare soil (UCLN pattern), and upper half non-crusted bare soil with lower half BSCs (UNLC pattern) (Fig 2). The two BSCs species, mosses and lichens, both were involved in the four mentioned micro-patterns, respectively. Their mean value in the same pattern was used to analyze the related hydrological response.

### Soil sampling and measurement

Following two years of natural succession and development, the cultivated BSCs species (mosses and lichens) had grown successfully and had become integrated with the topsoil. The crustal lichens appeared as a grey-black color that was tightly attached to the soil. The mosses formed a yellowish green cover (Fig 2). Mosses have highly-developed rhizoid systems and can fix soil particles tightly. Based on field measurements, the depth of the two BSC types was less than 10 cm of the topsoil. PCs, however, formed mainly by rainfall splatter. Next, we collected soil samples of the top 10 cm soil layers from the 4 supplemental micro-plots in July, 2013, using the cutting-ring method. Key parameters, including soil buck density, water holding capacity, total soil porosity, and capillary porosity were measured for further analysis.

### Infiltration and soil stability simulation

**Fig 3:**
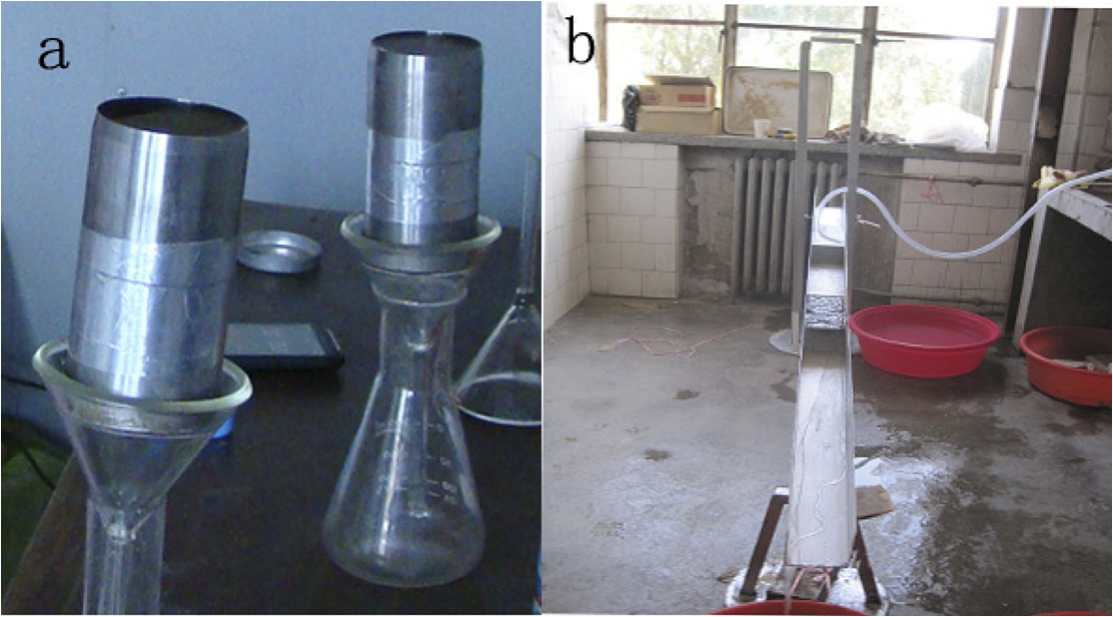
Infiltration simulation (a) and soil anti-scourability test (b).

To analyze the effect of BSCs on soil hydrologic attributes more systematically, water infiltration capacity and soil anti-scourability under different crusts (BSCs and PCs) and non-crusted bare soils were simulated. The specific infiltration process was simulated by using the method of "two cutting-rings" [33], where an empty cutting-ring was placed directly on another that was filled with crusted soils (Fig 3A). Waterproof tape was used to prevent leakage between the rings. The rings were kept vertical. A funnel and beaker were placed below the rings to collect the filtrated water. The rings were 5.1cm high with a 100 cm^3^ total volume. The initial drops of water were recorded at a 1 min interval during the first 10 min, and the recorded time interval was then changed to 5 min. All infiltration experiments were conducted for 60 min.

To further clarify the effect of different crusts on soil stability and erosion-resistance, additional soil anti-scourability experiments were conducted by using the "undisturbed soil washing box" method [34] (Fig 3B) with the box measuring 5cm (width) × 10 cm (depth) × 10 cm (length). In August, 2013, the same-sized crusts were sampled carefully from the 4 mentioned supplemental plots, and placed into the washing box undamaged for simulation. The washing flow rate was set at 2 m/l. The simulation lasted 60 min with data recorded every 5minutes. Surface runoff was measured by a graduated cylinder, while the collected sediments were dried in a baking oven and then weighed. The washing damage rate was estimated by visual observation.

### Rainfall-induced runoff and erosion simulation

Rainfall simulation experiments were conducted *in situ* during the growing season, i.e., from July to September in 2013. A portable rainfall simulator was used with a SPRACO cone jet nozzle, mounted 4.57 m above the micro-plots (Fig 2). This method has been used extensively in plot-scale hydrological studiesfor the data reliability and manipulation convenience [35–36]. The median volume of rain-drop size obtained by this simulator was approximately 2.4 mm. The kinetic energy of the simulated rainfall was approximately 90% of the natural rainfall. In most cases, the rainfall uniformity reached 89.70% during the whole simulation process, and the mean rainfall-intensity was designed as 0.50mm/min in less than 1 h. The average return-period of simulated rainfall in this study is calculated as once in a 0.87-year, through comparison with long-term rainfall data collected from local meteorological station.

To minimize the disturbance of the simulation by mountain breeze, we selected days with negligible wind. Runoff and soil loss was recorded at 5 min intervals. To compare the effect of the different micro-patterns of BSCs and PCs types on runoff and erosion, the total runoff volume and sediment yield after each simulated rainfall were collected and measured. Each rainfall simulation experiment was completed within 60 min.

In total, more than 120 rainfall simulations were conducted in the sampled micro-plots during the rainy season of 2013. During the simulation, each specific hydrological parameter was recorded, and field data were collected during the simulated time. When the experiments ceased, all data were re-checked carefully, and 84 rainfall simulations were selected for further analysis.

### Statistical analysis

The standard deviation of soil hydrologic attributes and runoff-erosion responses between different BSCs were conducted in SPSS16.0 for Windows. All histograms and line charts, depicting the features of soil hydrological responses, were drawn using Excel 2007.

## Results

### Soil physical and infiltration properties of different BSCs

BSCs play a key role in the improvement of soil hydrologic properties (Table 1). We found that mosses were most efficient in improving water holding capacity and soil porosity, followed by lichens and non-crusted bare soils. PCs reduced soil porosity compared with BSCs, and soil buck density decreased in the decreasing order of mosses, lichens, non-crusted bare soil, and PCs. Furthermore, under the influence of BSCs, the soil clay content increased slightly in the order of non-crusted bare soils, physical crusted soils, lichens and mosses. Significant difference of soil particle size distribution was observed between BSCs and PCs in most cases, while no significance was found between mosses and lichens, or between PCs and non-crusted bare soils. Other key parameters, such as soil capillary, water holding capacity, and capillary porosity, all changed in the similar order. The mean depth of topsoil crusts increased from PCs to lichens and mosses.

**Table 1:** Soil hydrologic characteristics under different surface soil crusts.

Soil crust type	Mean depth mm	Clay %	Silt %	Sand %	SBD g/cm ^3^	HWC %	CWC %	LWC %	TSP %	CSP %	NCP %
Mosses	15.7 ± 2.0	3.48a	68.05a	28.47a	1.08a	59.56a	47.22a	34.93a	61.64a	38.76a	14.85a
Lichens	10.6 ± 1.4	3.41a	67.47a	29.11a	1.21b	38.07b	30.64b	21.55b	50.34a	37.49a	9.27b
PCs	7.5 ± 0.9	3.28b	65.89b	31.41b	1.38c	29.85c	21.95c	14.37c	33.86b	29.58b	4.52c
non-crust	—	3.16b	64.49b	32.35b	1.31d	31.15c	23.09d	16.67c	44.07c	27.65b	5.94c

SBD—soil buck density, HWC—highest water holding capacity, CWC—soil capillary water holding capacity, LWC- lowest water holding capacity, TSP—total soil porosity, CSP—soil capillary porosity, and NCP—non-capillary porosity. Different letters (a, b and c) in the same rows indicate that the significant statistically difference between each two crusts.

**Fig 4:**
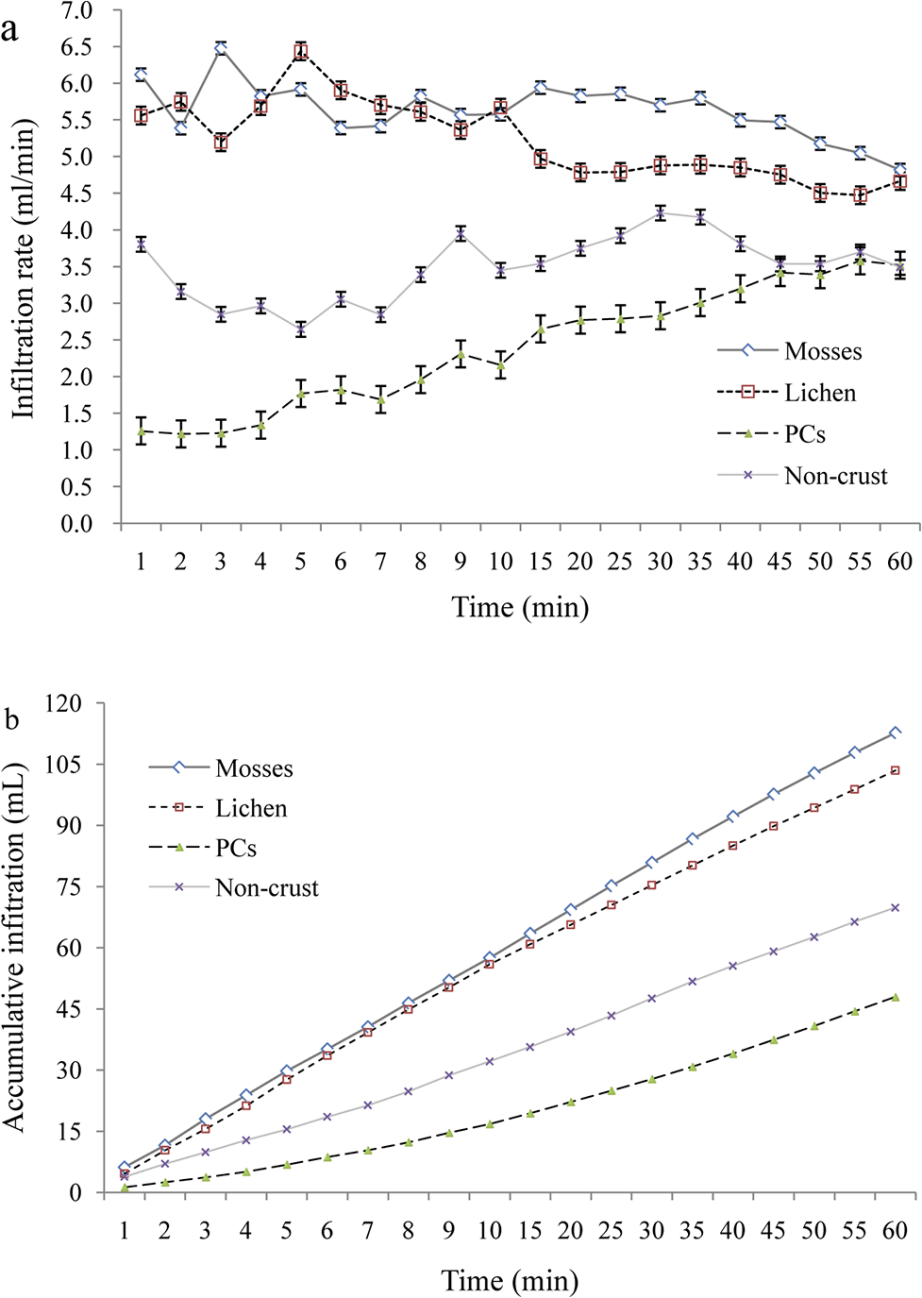
Infiltration process under different BSCs and non-BSCs soil types.

Results show that the specific infiltration process under different BSCs varied greatly during the simulated 60 minutes (Fig 4). BSCs appeared to be more effective in increasing the rate and total amount of soil infiltration compared with non-crusted soils and PCs. During the complete simulation time, the infiltration capacity under mosses was higher than that under lichens. Moreover, it was found that the infiltration capacity of PCs remained relatively stable during the first minutes, then gradually increasing, until finally achieving a similar infiltration rate as the non-crusted bare soils (Fig 4A). The infiltration capacities under BSCs (mosses and lichens) remained relatively stable during the first 10–15 minutes, before decreasing. The total infiltration capacity was decreased in the order of mosses, lichens, non-crusted bare soil and PCs (Fig 4B).

### Runoff and soil loss under different BSC species

**Fig 5:**
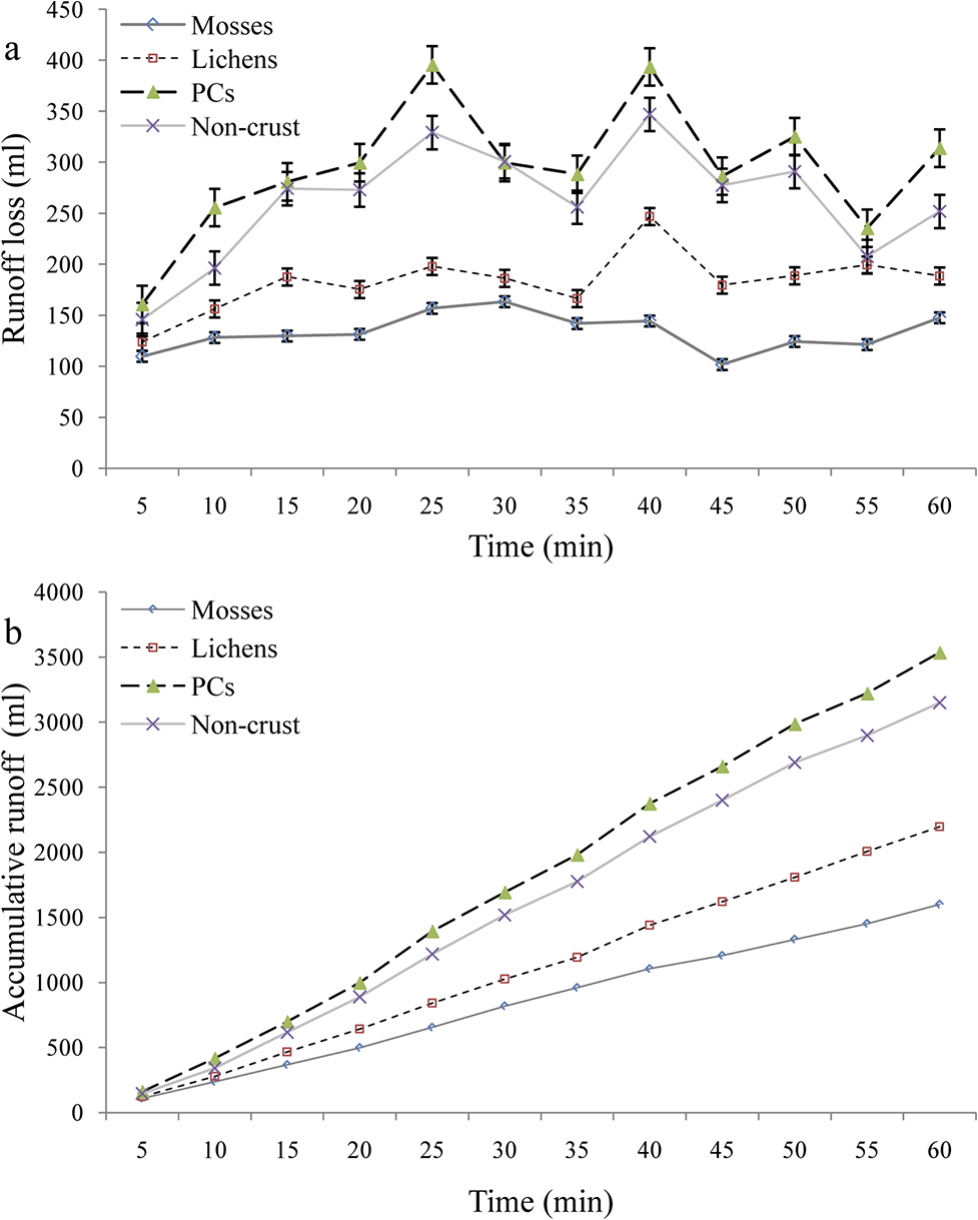
Runoff process under different types of fully-covered surface crust.

The specific process of runoff generation was captured by the rainfall simulation experiments *in situ* (Figs 2 and 5). In general, surface runoff under the different crusted and non-crusted soils differed significantly. Mosses had the least runoff loss, followed by lichens and non-crusted bare soils, whereas PCs had the highest surface runoff loss. This indicates that BSCs are more effective in reducing runoff than non-crusted bare soil, while PCs can generate runoff due to their poor infiltration and water holding capacities (Table 1). The runoff for mosses and lichens appeared more stable across the simulation period. The runoff process for PCs and non-crusted bare soils, on the other hand, varied significantly during the simulated 60 minutes (Fig 5A). The rates of cumulative runoff under PCs and non-crusted soils were similar during the simulation, while the values for mosses and lichens were closer to each other (Fig 5B).

**Fig 6:**
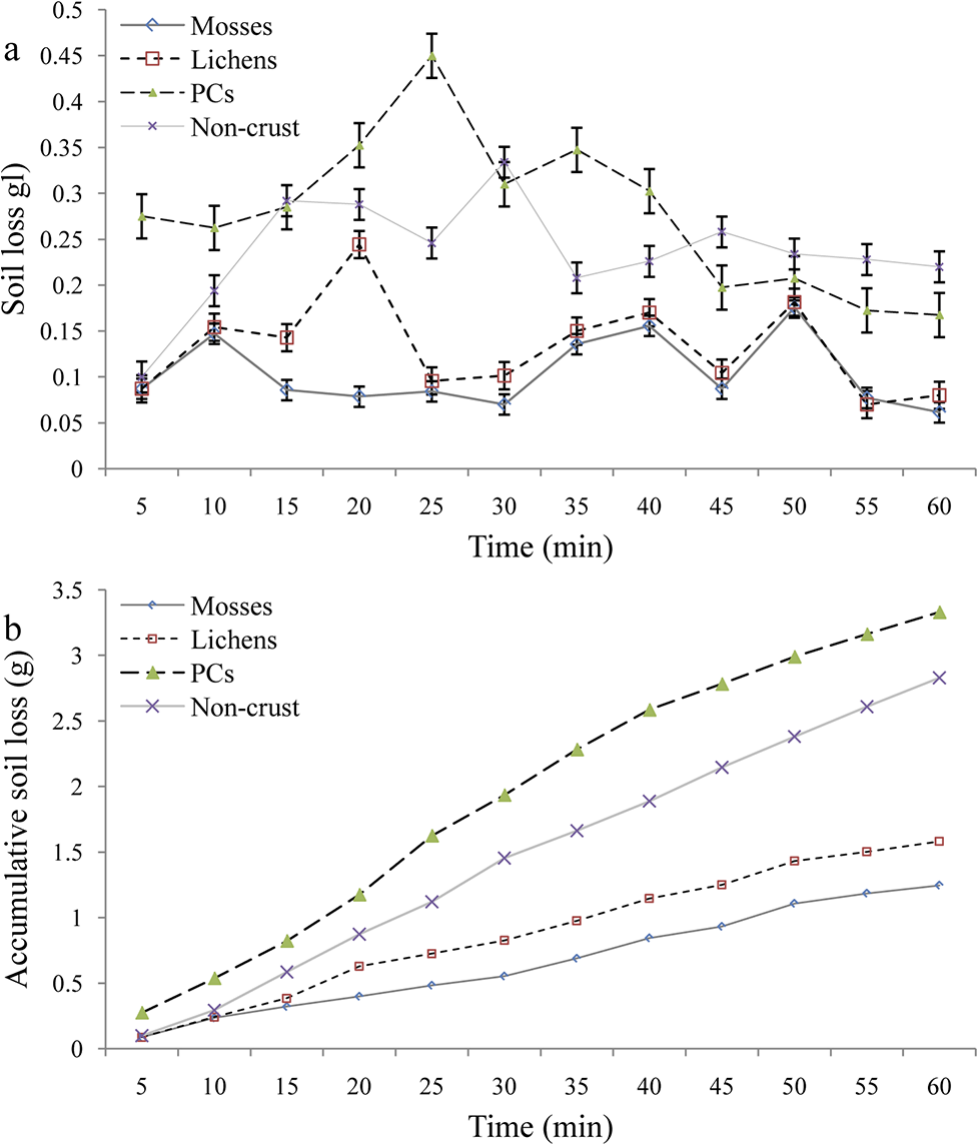
Soil loss process under various BSCs types.

The soil losses under different crusts and non-crusted bare soils were also analyzed (Fig 6). For mosses, lichens and non-crusted soils, the corresponding sediment varied within the simulated time, but no clear trend (increase or decrease) was observed (Fig 6A). For PCs, however, we found that soil loss during the first 15 min remained stable, then increased sharply and reached the maximum value during approximately the 25th min interval. From here, soil loss decline gradually, reaching values lower than those in the non-crusted bare soil after 40 min. In total, the amount of soil loss increased from mosses, to lichens, non-crusted bare soils and PCs (Fig 6B).

### Runoff and soil loss under different BSC micro-patterns

**Fig 7:**
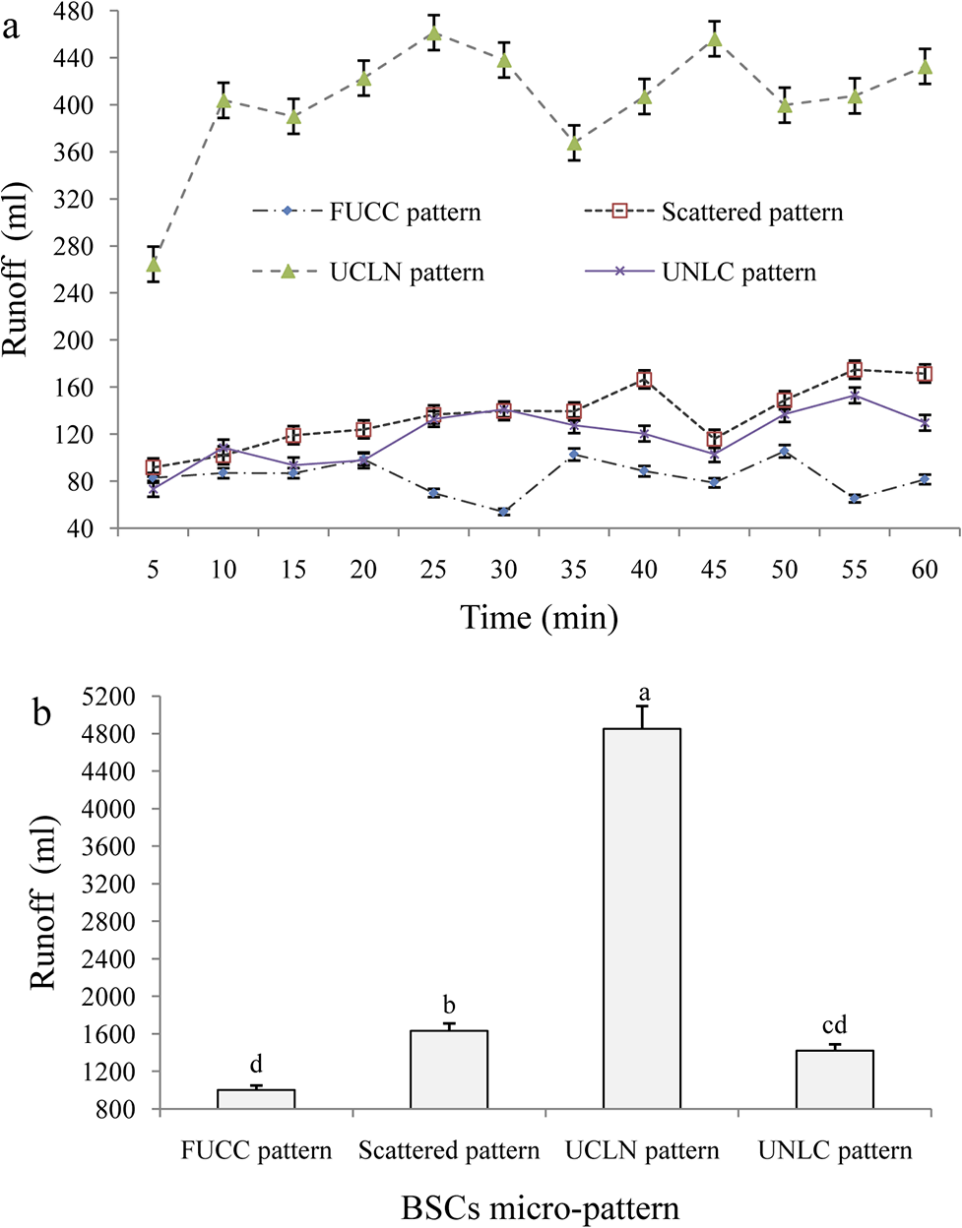
Runoff losses under different micro-patterns of BSCs. (Note: FUCC pattern, Scattered pattern, UCLN pattern and UNLC pattern refers to fully-covered BSCs pattern, scattered BSCs pattern with 50% BSC coverage, upper half BSCs with lower half non-crusted bare soil pattern, and upper half non-crusted bare soil with lower half BSCs pattern, respectively.)

The dynamics of soil and water loss under the four major micro-patterns of BSCs were analyzed by using rainfall simulation method (Fig 7). The values under each pattern were calculated by the means of the corresponding moss and lichen patterns. We found that the fully-covered BSC pattern (FUCC) was best in controlling runoff loss, followed by the scatter-covered BSC and UNLC (upper non-crust and lower crust) patterns (Fig 7A). The UCLN (upper crust and lower non-crust) pattern, however, experienced the most severe runoff loss, which was higher than that under the other three crusted patterns. Furthermore, the significance test of variance showed a marked difference may exist between the FUCC and UCLN patterns. Although there was a difference in actual values between the scattered and UNLC patterns, it was not statistically significant (Fig 7B).

**Fig 8:**
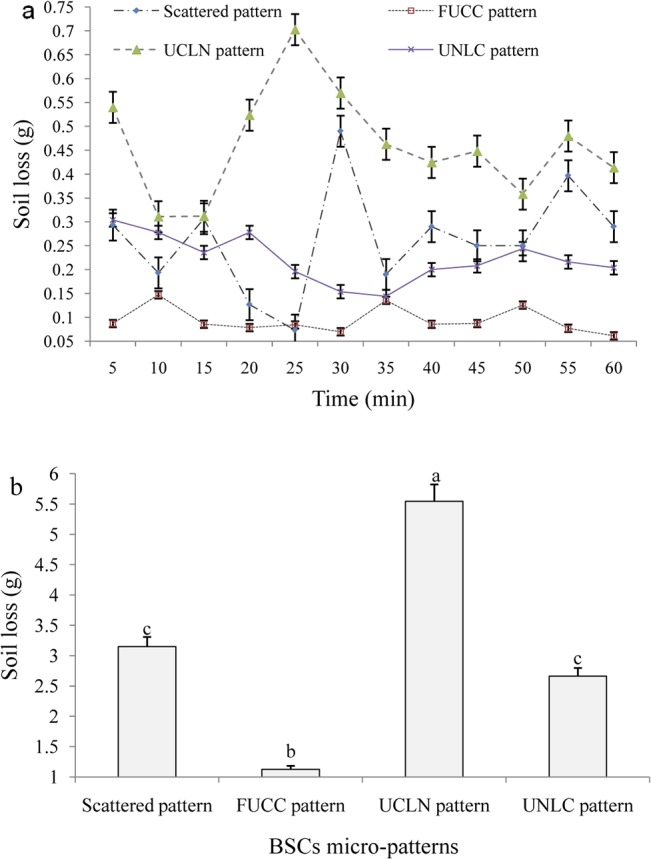
Soil losses under different BSC micro-patterns. (Note: FUCC pattern, Scattered pattern, UCLN pattern and UNLC pattern refers to fully-covered BSCs pattern, scattered BSCs pattern with 50% BSC coverage, upper half BSCs with lower half non-crusted bare soil pattern, and upper half non-crusted bare soil with lower half BSCs pattern, respectively.)

The related dynamics in soil loss during each simulated rainfall event was also recorded and analyzed. Results show that the least soil loss occurred under the FUCC pattern during the simulation period, which was much lower than that under other micro-patterns (Fig 8). Soil loss under the remaining three micro-patterns increased in an order of UNLC, scattered and UCLN patterns. We also found that soil loss under scattered and UCLN patterns varied more than under FUCC and UNLC patterns across the simulation time (Fig 8A). Significant difference of variance was found among FUCC, UCLN and UNLC patterns, while the difference between scattered and UNLC patterns was not significant (Fig 8B).

## Discussion

### Effect of BSCs types on soil permeability

Our study suggests that topsoil BSCs, such as mosses and lichens, can effectively improve soil hydrologic properties at the first 5–10 cm layers (Table 1 and Fig 4). Our experiments re-confirmed that mosses and lichens functioned better than PCs by decreasing soil buck density, increasing water holding capacity and infiltrability. This conclusion is consistent with other researches [14, 37]. One possible reason for this is that mosses may have higher biomass (Table 1) than lichens. Meanwhile, some studies also found that the surface roughness (micro-relief) under mosses was higher than lichens [23]. Higher roughness can delay the time of water flow, and thus benefit infiltration and improve soil water content [27]. Furthermore, mosses are considered to be a higher stage of succession than lichens, being more effective in changing soil structure and biochemical characteristics [37]. Other studies have also indicated that the formation and temporal dynamics of BSCs are responsible for the spatial variability of soil infiltration capacity and other hydrologic attributes [10, 13, 20, 31, 38]. Based on soil moisture measurements before and after rainfall simulations, Xiao et al. [12] confirmed that cultured BSCs (mainly mosses) can increase infiltration and improve soil water content. Belnap et al. [26] also pointed out that the specific characteristics of surface BSCs can determine the amount, location and timing of water infiltration into desert soils.

PCs at surface soil layer, however, may have double effects on soil hydrology. One the one hand, it is inclined to increases runoff which would increase erosion, but on the other it increases soil surface resistance which may reduce particle detachment. In this study, PCs was found to decrease soil aeration and porosity (Table 1), and increase soil penetration-resistance (Fig 4). Similar phenomena were also found in other studies. For examples, compared with the BSC species, PCs generally have lower hydraulic conductivity and infiltration rates [39]. Li [9] found that the total amount of rainwater infiltration can be reduced by over 90% because of abiotic crusting induced by raindrop or runoff splashing, particularly at the beginning of rainfall simulations. PCs are often defined as key symbols of land-degradation and desertification [5, 9, 18, 39–41]. Loess soil will readily generate more runoff, often as a result of the formation of PCs in the desert soils [42], although BSCs may develop from PCs [18].

In this study, non-crusted bare soils can promote soil infiltration better than PCs, but less than BSCs (Table 1 and Fig 3). Similar results were also found by other studies. Infiltration rates of sandy soils in Mali, for example, were observed to range from 100 to 200 mm hr^-1^ without crusts, but reduced to 10 mm hr^-1^ after PCs formation [43]. Before and after PCs, infiltration rates in Israel were reduced from 100 mm hr^-1^ to 8 mm hr^-1^ in sandy soils, and from 45 mm hr^-1^ to 5 mm hr^-1^ on a loess soil [44]. Reduced permeability, however, can significantly decrease the water available for local plant growth [6, 9].

### Effect of BSCs species on water erosion and soil stability

**Fig 9:**
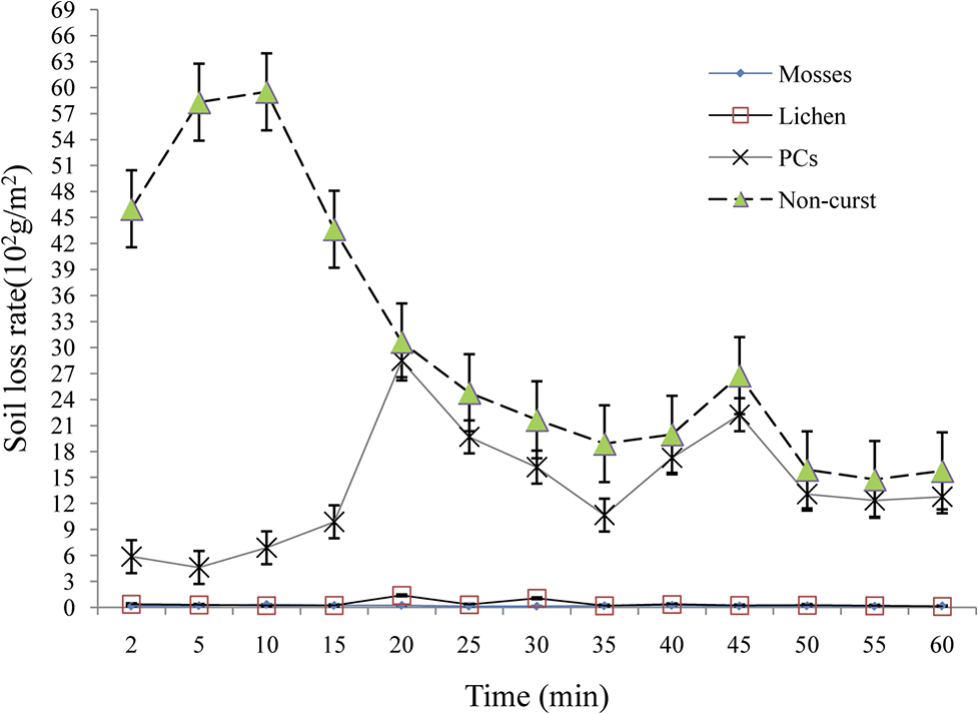
Soil anti-scourability under BSCs and non-BSCs conditions.

We found that the BSCs species (mosses and lichens) performed a more effective role in runoff retention and soil loss control than PCs and non-crusted bare soils (Fig 5). Mosses function better than lichens, followed by non-crusted and PCs. This indicates that the types and succession stages of different BSCs play key roles in altering sediment and water redistribution in the arid and semiarid ecosystems. PCs, however, are poorer than non-crusted soil surfaces in minimizing the consequences of water erosion. Furthermore, our additional soil stability test also confirmed that BSCs are better at stabilizing topsoil structures and resisting water erosion than PCs or non-crusted bare soils (Figs 3 and 9; Table 2).

**Table 2:** Mean soil anti-scourability under the same washing-flow rate (2 L/min).

Soil crust type	Mean erosion rate (g/m^2^)	Surface destroy ratio(%)
**Mosses**	27.1	0.30
**Lichens**	54.1	0.61
**PCs**	1800.3	21.55
**Non-curst**	3915.7	—

These findings are consistent with other studies conducted in different climatic regions [24, 45]. For example, some studies have concluded that BSCs can alter micro-topography and increase surface roughness at fine scales, and suggest a major contribution to runoff reduction [11, 46]. In general, BSCs can substantially reduce soil erosion caused by water or wind [15, 47]. Whisenant [6] also concluded that soil surface stability is most improved in locations with abundant lichens and mosses. In SE Australia and Utach, North America, lichens and mosses can fix soil particles in the top centimeters of surface soil, mainly through their penetrating rhizines [48–49]. Filamentous cyanobacteria in the Tegger Desert in China can exude polysaccharides, which benefit soil aggregate formation and enhance soil stability, finally contributing to runoff retention and erosion control [9]. The cohesion of lichens, cell density, and cyanobacteria biomass are more pertinent for soil stabilization and fertility in the degraded ecosystems [15]. Accordingly, this can be mainly attributed to the interaction between soil physiochemical structure and microphytic community of BSCs. Such microorganisms secrete mucilaginous polysaccharide sheaths that bind soil particles more tightly [50–51]. Consequently, it can help to stabilize surface soil against water erosion. Moreover, as mosses are part of the higher stages of primary succession, they can accumulate more biomass and develop more advanced rootstock systems for fixing soil particles [37]. This may help explain why mosses are better than lichens in reducing water erosion rates.

### Effect of BSCs coverage on runoff generation and soil loss

Our study indicates that the surface coverage of BSCs can markedly alter the specific process and consequence of runoff generation and soil loss. Based on our results, the mean values of runoff and soil loss under fully-covered BSCs were far lower than those under half-covered BSCs, respectively (Figs 6 & 7). This indicates that an effective surface cover of BSCs will act as a powerful sink for runoff and sediment yield. Uncovered bare soil and PCs conditions, however, are strong sources of soil and water loss. The greater the coverage by BSCs, the lower the potential risk and actual yield of runoff and soil erosion will be.

Similar conclusions were also found in other regions. In eastern Australia, for example, soil surfaces with higher coverage of BSCs were more stable and less prone to erosion than the soil surfaces with less BSC cover [6, 50]. Another study also pointed out that when the total coverage of mosses decreased from 74.4% to 5.0%, organic matter content declined from 9.35 to 1.71 g/kg and soil buck density increased from 1.35 to 1.54 g/cm^3^ [9]. Such degradation in soil hydrologic attributes will in turn certainly increase the potential for runoff and soil erosion [18, 30]. Although some studies have pointed out that the surface protection of BSC coverage is weaker than the coupling effects of BSCs and grass /shrub communities [33, 52], it is still better than non-crusted bare soil (with 0% BSCs coverage) conditions (Figs 4 & 5). In such cases, to avoid higher potential water erosion, other additional measures (e.g., re-vegetation or surface mulching using different materials) are strongly suggested when the BSC coverage has abruptly reduced.

### Effects of micro-pattern of BSCs on runoff and soil loss

This study has confirmed that the spatial distribution and specific position of BSCs, thought to be one of the key soil surface components in the arid and semiarid ecosystems [30,37,53], play dominant roles in the magnitude of runoff and soil loss on non-vegetated landscape surfaces (Figs 2, 6 & 7). In general, BSC patches clearly reduce runoff and sediment production, while bare soil patches act as strong sources of soil and water loss at micro-plot scales. Based on our results, the fully-covered BSC pattern should provide the most runoff retention, followed by the scatter-covered BSC and UNLC (upper bare soil and lower BSCs), and finally UCLN (upper BSC and lower non-crusted bare soil) patterns (Fig 6). However, the UNLC performs better than the scattered BSC pattern in reducing soil loss, followed by the UCLN pattern. No significant difference was found between the scattered and UNLC patterns (Fig 7). This means that the spatial position of surface BSCs in the micro-plots is significant for runoff generation and sediment transport. When the BSCs are in the upper position of the plots with bare soils at the bottom, the most soil loss occurs. Conversely, BSCs located in the lower position of the plots acted as an effective buffer in reducing runoff and soil loss.

As explained above, the two-phase mosaic (BSC and bare soil) pattern is crucial in regulating water erosion rates [12, 30]. When the surface coverage is same or similar (e.g., approximately 50% BSCs in this study), how to manage the distribution of BSCs, or other types of higher vegetation, will become most significant. This means that the spatial distribution of soil surface components will greatly affect the surface soil condition and hydrological behavior [53], and this interesting topic has resulted in several studies around the world. For example, the distribution of BSC morpho-types in Mexico were confirmed to be responsible for determining soil stability, which directly influence the total output of sediment and soil loss [15]. A mosaic or pattern containing BSCs and soil patches bare of vegetation, distributed over hill-slopes either grouped together or at random, can dominate the generation of runoff or infiltrate surface flow [54–55]. In the loess hill areas, we also found that runoff loss control is most effective when the shrub grows in the lower part of the plot, followed by shrub in the middle and shrub in the upper positions [52].

## Conclusion and Suggestions

In this study, the type and spatial pattern of BSCs were studied at a micro-plot scale in a loess hilly area in China. Based on hydrological simulations, the role of two major BSCs (mosses and lichens) in soil attributes, infiltration capacity, and water erosion consequence was compared with PCs and non-crusted bare soils. Generally, mosses and lichens are confirmed more effective than non-crusted soils and PCs at infiltration enhancement, runoff retention and erosion control. Mosses functioned better than lichens, likely due to the higher succession stage of mosses, while PCs had a negative influence on soil hydrologic properties. Fully-covered mosses and lichens were confirmed to perform better than half-covered BSCs in water erosion reduction. The upper non-crusted bare soil and lower BSCs patterns, as well as scatter-covered BSC patterns were more effective than upper BSCs with lower non-crusted bare soil pattern in controlling water erosion.

These findings may prove useful, particularly in the water-limited ecosystems and degraded slopes. For example, the development of BSCs is encouraged in the non-vegetation covered areas because they are strong sinks for infiltration-runoff and aid soil-water conservation. More importantly, great attention should be paid to the spatial arrangement and distribution features of BSCs across the slopes. When some disturbances (deforestation, overgrazing and trampling) are unavoidable in practice, we strongly suggest minimizing the destruction of BSCs in the lower/bottom positions, instead choosing the upper positions of the slopes. It is better to carry out cultivated BSCs (e.g., mosses) and other essential countermeasures (e.g., re-vegetation projects and various surface mulching techniques) in the disturbed areas, targeting increasing the surface coverage and reducing water erosion hazards.
